# Defining cognitive impairment in people-living-with-HIV: the POPPY study

**DOI:** 10.1186/s12879-016-1970-8

**Published:** 2016-10-28

**Authors:** Davide De Francesco, Jonathan Underwood, Frank A. Post, Jaime H. Vera, Ian Williams, Marta Boffito, Memory Sachikonye, Jane Anderson, Patrick W. G. Mallon, Alan Winston, Caroline A. Sabin, on behalf of the POPPY study group

**Affiliations:** 1 Research Department of Infection & Population Health, UCL - Royal Free Campus, London, UK; 2 Division of Infectious Diseases, Imperial College London, London, UK; 3 King’s College London, London, UK; 4 Brighton and Sussex Medical School, Brighton, UK; 5 Mortimer Market Centre, UCL, London, UK; 6 Chelsea and Westminster Healthcare NHS Foundation Trust, London, UK; 7 UK Community Advisory Board, London, UK; 8 Homerton University Hospital, London, UK; 9 UCD School Of Medicine, Dublin, Ireland

**Keywords:** HIV, Cognitive impairment, Patient-reported cognitive symptoms, Neurology, HIV-associated neurocognitive disorder

## Abstract

**Background:**

The reported prevalence of cognitive impairment (CI) varies widely in cohorts of people living with HIV (PLWH); this may partly be due to the use of different diagnostic criteria. Agreement between diagnostic criteria of CI, the optimal definition to use, and associations with patient-reported cognitive symptoms have not been fully investigated.

**Methods:**

Two hundred ninety PLWH aged >50 years and 97 matched negative controls completed a detailed assessment of cognitive function and three questions regarding cognitive symptoms. Age- and education-adjusted test scores (T-scores) determined if subjects met the following definitions of CI: Frascati, global deficit score (GDS) and the multivariate normative comparison (MNC) method.

**Results:**

PLWH were more likely than controls to meet each definition of CI (ORs were 2.17, 3.12 and 3.64 for Frascati, GDS and MNC, respectively). Agreement of MNC with Frascati and GDS was moderate (Cohen’s *k* = 0.42 and 0.48, respectively), whereas that between Frascati and GDS was good (*k* = 0.74). A significant association was found between all the three criteria and reporting of memory loss but not with attention and reasoning problems. The 41 (14 %) PLWH meeting all the three criteria had the lowest median global T-score (36.9) and highest rate of symptom reporting (42 %).

**Conclusions:**

Different CI criteria show fair diagnostic agreement, likely reflecting their ability to exclude CI in the same group of individuals. Given the lower overall cognitive performance and higher rates of symptom reporting in those meeting all three criteria of CI, further work assessing this as a definition of CI in PLWH is justified.

**Electronic supplementary material:**

The online version of this article (doi:10.1186/s12879-016-1970-8) contains supplementary material, which is available to authorized users.

## Background

In recent years, despite the introduction of combination antiretroviral therapy (ART), a high but varying prevalence of cognitive impairment (CI) has been reported among HIV-positive individuals. Robertson et al. [1] described a 26 % prevalence of CI among HIV-positive subjects who had received ART for at least 20 weeks, while Heaton et al. [2] reported a prevalence of 36 % among ART-treated asymptomatic individuals. Among those with long-standing viral suppression, Winston et al. [3] and Simioni et al. [4] reported prevalences of 51 and 74 %, respectively.

The optimal screening tools to identify CI are unknown. The current European AIDS Clinical Society (EACS) guidelines recommend three questions as one form of assessment to guide the initial evaluation of HIV-positive individuals with suspected cognitive problems [5]. These patient-reported measures may be a quick and practical method of screening for cognitive impairment in clinical practice, however, doubts about their utility remain [6]. When cognitive function is assessed through objective neuropsychological tests, differences in the reported prevalence of CI may be due to the use of different diagnostic criteria. Three criteria in current use include the ‘Frascati’ criteria, proposed by Antinori et al. [7] and also known as HIV-associated neurocognitive disorder (HAND) criteria, the global deficit score (GDS) [8] and the multivariate normative comparison (MNC) [9]. These criteria differ in the way they combine scores from a battery of neuropsychological tests to classify subjects as either cognitively impaired or normally functioning. Several studies have reported contrasting results when using different criteria of CI on the same set of patients [10, 11]. However, they only assessed prevalence rates by criteria and did not specifically investigate whether they identified the same people as impaired/not impaired.

The associations between CI and patient-reported outcomes of cognitive function have not been fully investigated and remain unclear. Some studies have found a relationship between subjective cognitive complaints and actual impairment in neuropsychological tests [12, 13], while other studies have not [14, 15]. While these studies tended to include high rates of untreated or unsuppressed subjects, there is currently a lack of data relating cognitive function with self-reported cognitive complaints in populations of well-treated HIV-positive individuals.

The aims of this study are threefold. Firstly, to compare the prevalence of CI in HIV-positive individuals over 50 years of age and demographically matched HIV-negative controls according to the Frascati criteria, GDS and MNC and combinations of these definitions. Secondly, to assess the level of agreement between these three criteria when identifying HIV-positive people with CI. Finally, to investigate the association between different definitions of CI and their combination with patient-reported symptoms of cognitive dysfunction.

## Methods

### Study design and participants

The Pharmacokinetic and Clinical Observations in People Over Fifty (POPPY) study is a prospective, multicentre, observational study that aims to examine the effects of ageing on the clinical outcomes of people living with HIV in UK and Ireland. To address its aims the study has established cohorts of HIV-positive people aged over 50, younger HIV-positive controls less than 50 years old and demographically matched HIV-negative controls aged over 50 years. For the present analysis only the two older cohorts were considered as the purpose of the younger HIV-positive cohort is to provide a younger control group which is not directly relevant to this analysis. HIV-positive participants were recruited at HIV outpatient clinics around UK and Ireland. Inclusion criteria were: documented presence of HIV infection, self-defined white or black-African ethnicity, likely route of HIV acquisition via sexual exposure (either by male to male exposure if white or by heterosexual exposure if white or black-African) and ability to comprehend the study patient information leaflet. HIV-negative controls were frequency matched to the HIV-positive group on gender, ethnicity, sexual orientation and location (in or out of London) and were recruited from sexual health clinics affiliated with the HIV clinics, as well as from community events, churches, adverts in targeted publications and community groups. Recruitment was from January 2013 to September 2014. The study was approved by the UK National Research Ethics Service (NRES; Fulham London, UK number 12/LO/1409). Written informed consent was obtained from all participants prior to undertaking any study specific procedures.

### Cognitive symptoms

All enrolled participants completed questionnaires detailing physical and mental health status. In particular, participants answered the three questions on cognitive symptoms described by the EACS guidelines [5] regarding memory loss (do you experience frequent memory loss [e.g., do you forget the occurrence of special events even the more recent ones, appointments, etc.]?), reasoning (do you feel that you are slower when reasoning, planning activities, or solving problems?) and attention (do you have difficulties paying attention [e.g., to a conversation, book or movie]?). Individuals answering ‘Yes, definitely’, as opposed to ‘Never’ and ‘Hardly ever’ were classified as experiencing the related cognitive symptom. In addition a positive answer to at least two of the three questions was considered indicative of self-reported cognitive problems.

### Assessment of cognitive function

Assessment of cognitive function was performed using the CogState battery [16], a computerized battery of neuropsychological tests that has been used in different clinical settings [17–20], including HIV-positive cohorts [21–24]. The battery covered six cognitive domains commonly affected by HIV-associated CI, including visual learning, psychomotor function, visual attention, executive function, verbal learning and working memory (see Additional file 1: Table S1 for details of individual tests and how they map onto cognitive domains). Raw test scores were log-transformed or arcsine root–transformed where necessary (as recommended by the CogState guidelines for analysis) and converted into demographically-adjusted T scores (with a mean of 50 and a standard deviation of 10) using the scores of the HIV-negative group as normative scores. Briefly, a linear regression was fit for each test in order to estimate regression coefficients for age, gender, ethnicity and education using scores from the HIV-negative group. These regression coefficients were then used to determine the normative scores depending on subjects’ age, gender, ethnicity and education. The difference between the normative score and the actual score for each subject was then standardized into T-scores. A single T-score was calculated for each of the 6 cognitive domains by averaging individual test T-scores within each domain. A global T-score was also obtained by averaging T-scores across the six domains. For all T-scores a higher value indicates better cognitive function.

### Classification of CI

For each subject, the T-scores were then used to determine if the individuals met three definitions of CI, Frascati, GDS and MNC, using published methods. Frascati and GDS are the most extensively adopted definitions in previous studies of CI in HIV, while the MNC is a relatively newer approach showing promising results in reducing the false positive rate [10]. First, according to the Frascati criteria [7], CI was defined as at least two cognitive domain T-scores below 40 (i.e., one or more standard deviations below the average normative score). Second, a GDS [8] was computed for each subject by converting domain T-scores into deficit scores (0: T-score ≥ 40, 1: 34 < T-score < 40, 2: 29 < T-score ≤ 34, 3: 24 < T-score ≤ 29, 4: 19 < T-score ≤ 24, 5: T-score ≤ 19). An overall GDS was obtained by averaging domain deficit scores and CI was defined as a mean score equal or greater than 0.5. Finally, the MNC method [9] was applied. The MNC is a statistical method that simultaneously compares multiple cognitive scores of each study participant to the average scores of the same tests in the control group (in our case the HIV-negative group), taking into account the variances and covariance between all scores. For each participant, a continuous measure of the deviation of the participant’s cognitive profile from the average cognitive profile in the control group is then obtained. If this deviation (also called Hotelling’s T^2^) exceeds a critical value associated with a 5 % significance the individual is classified as cognitively impaired (so that the chance of erroneously concluding that an individual has CI while this is not the case, i.e., the false positive rate, is approximately 5 %). Definitions of CI included the three criteria listed above plus all combinations of patients meeting individual criteria and two or all three of these criteria.

### Statistical analysis

Group comparisons of baseline characteristics were assessed using Chi-square, Wilcoxon rank-sum and t-tests (two-tailed) as appropriate. Comparisons of the prevalence of CI in HIV-positive and HIV-negative persons were performed using the Chi-square test with odds ratios used to provide a comparative estimate of the prevalence in the two groups. The agreement between criteria of CI was assessed using Cohen’s κ statistics [25] and interpreted following Landis and Koch [26] guidelines. The null hypothesis that Cohen’s κ equals zero (i.e., no agreement between criteria other than what would be expected by chance) was tested using the asymptotic test [27]. Internal consistency between the three patient-reported cognitive problems was assessed using Cronbach’s α. Associations between each definition of CI and self-reported cognitive problems were assessed using the Chi-square test. All analyses were performed using SAS v9.4 with *p*-values <0.05 considered as statistically significant.

## Results

### Participant characteristics

A total of 290 HIV-positive and 97 HIV-negative participants were enrolled into the study between January 2013 and September 2014 and completed the CogState battery. Demographic, lifestyle and HIV-related characteristics are summarised in Table 1. Groups were highly comparable in terms of age (median age [IQR] was 57 [53, 62] and 58 [54, 62] years in HIV-positive and HIV-negative participants, respectively; *p* = 0.22), ethnicity (*p* = 0.47), country of birth (*p* = 0.92), educational attainment (*p* = 0.14), alcohol consumption (*p* = 0.13) and smoking status (*p* = 0.79). HIV-positive participants were more likely to be male (88.3 % vs. 66.0 %, *p* < 0.01), gay or homosexual (71.7 % vs. 41.2 %, *p* < 0.01) and to have reported recreational drug use in the 6 months preceding study entry (27.9 % vs. 12.4 %, *p* < 0.01) compared to HIV-negative controls. HIV-positives had been diagnosed with HIV for a median (IQR) of 16.8 (10.2, 22.9) years previously, and around 96.9 % were on ART with a median (IQR) CD4^+^ cell count of 610 (478, 780) cells/μL.

**Table 1:** Demographic, lifestyle and HIV-related characteristics of HIV-positive and HIV-negative study participants at enrolment (IQR: interquartile range)

	≥50 HIV-positive (*N* = 290)	≥50 HIV-negative (*N* = 97)	*p*-value
Age [years], median (range)	57 (50, 82)	58 (50, 83)	0.22
Gender, n (%)			<0.01
Female	34 (11.7 %)	33 (34.0 %)	
Male	256 (88.3 %)	64 (66.0 %)	
Ethnicity, n (%)			0.47
Black-African	37 (12.8 %)	9 (9.3 %)	
White	253 (87.2 %)	88 (90.7 %)	
Country of birth, n (%)			0.92
UK/Ireland	202 (69.7 %)	70 (72.2 %)	
Rest of Europe	17 (5.9 %)	4 (4.1 %)	
Africa	44 (15.2 %)	14 (14.4 %)	
Rest of the world	27 (9.3 %)	9 (9.3 %)	
Sexual orientation, n (%)			<0.01
Gay/Homosexual	208 (71.7 %)	40 (41.2 %)	
Bisexual	11 (3.8 %)	1 (1.0 %)	
Straight/Heterosexual	56 (19.3 %)	50 (51.6 %)	
Other/Unknown	15 (5.2 %)	6 (6.2 %)	
Education, n (%)			0.14
No qualification	31 (10.7 %)	4 (4.1 %)	
O levels/GCSEs (or equivalent at age 16)	41 (14.1 %)	18 (18.6 %)	
A levels (or equivalent at age 18)	41 (14.1 %)	20 (20.6 %)	
University degree or above	127 (43.8 %)	42 (43.3 %)	
Other/Unknown	50 (17.2 %)	13 (13.4 %)	
Smoking status, n (%)			0.79
Current smoker	70 (24.1 %)	20 (20.6 %)	
Ex-smoker	108 (37.2 %)	37 (38.1 %)	
Never smoked	111 (38.3 %)	39 (40.2 %)	
Not known	1 (0.3 %)	1 (1.0 %)	
Years of smoking (current/past smokers), median (IQR)	32 (20, 40)	33 (21, 40)	0.86
Alcohol consumption, n (%)			0.13
Current consumption	227 (78.3 %)	83 (85.6 %)	
Previous consumption only	40 (13.8 %)	6 (6.2 %)	
Never consumed alcohol	23 (7.9 %)	8 (8.3 %)	
Recreational drugs in past 6 months, n (%)	83 (27.9 %)	12 (12.4 %)	<0.01
Route of HIV-acquisition, n (%)			
Sex between men and women	61 (21.0 %)	N/A	
Sex between men	229 (79.0 %)	N/A	
Years since HIV diagnosis, median (IQR)	16.8 (10.2, 22.9)	N/A	
CD4^+^ cell count at enrolment [cells/μL], median (IQR)	610 (478, 780)	N/A	
On antiretroviral treatment, n (%)	281 (96.9 %)	N/A	
Viral load <50 copies/mL, n (%)	263 (90.7 %)	N/A	

### Cognitive test results and prevalence of CI

Overall performance of HIV-positive subjects was poorer than HIV-negative controls with a median (IQR) global T-score of 48.6 (43.5, 52.3) compared to 50.8 (46.0, 55.7) for the controls (*p* < 0.01). Significantly lower scores in the HIV-positive group, as compared to the HIV-negative group, were found for the cognitive domains: psychomotor function (median [IQR]: 48.4 [40.5, 54.4] vs 50.8 [45.4, 57.0], *p* < 0.01), visual attention (48.6 [39.7, 55.0] vs 50.9 [44.5, 57.0], *p* = 0.03) and verbal learning (47.0 [38.9, 53.6] vs 51.6 [45.3, 57.2], *p* < 0.01). In contrast, no significant group difference was found for the cognitive domains of visual learning (49.3 [42.7, 54.0] vs 50.8 [46.0, 55.7], *p* = 0.06), executive function (50.5 [45.9, 55.4] vs 50.4 [46.2, 54.8], *p* = 0.83) and working memory (50.1 [43.9, 54.4] vs 51.4 [46.0, 54.9], *p* = 0.15). The prevalence of CI in the HIV-positive group varied from 34.5 % according to GDS, 30.0 % according to Frascati and 22.1 % for the MNC. Similarly, the prevalence of CI varied in the HIV-negative group from 14.4 % (GDS) to 16.5 % (Frascati) and 7.2 % (MNC). According to all the three criteria, HIV-positive participants were significantly more likely of having CI than HIV-negative controls; OR (95 % CI) were 2.17 (1.20–3.92, *p* = 0.01) for Frascati, 3.12 (1.69–5.78, *p* < 0.01) for GDS and 3.64 (1.61–8.24, *p* < 0.01) for MNC.

### Agreement between definitions of CI

**Fig. 1:**
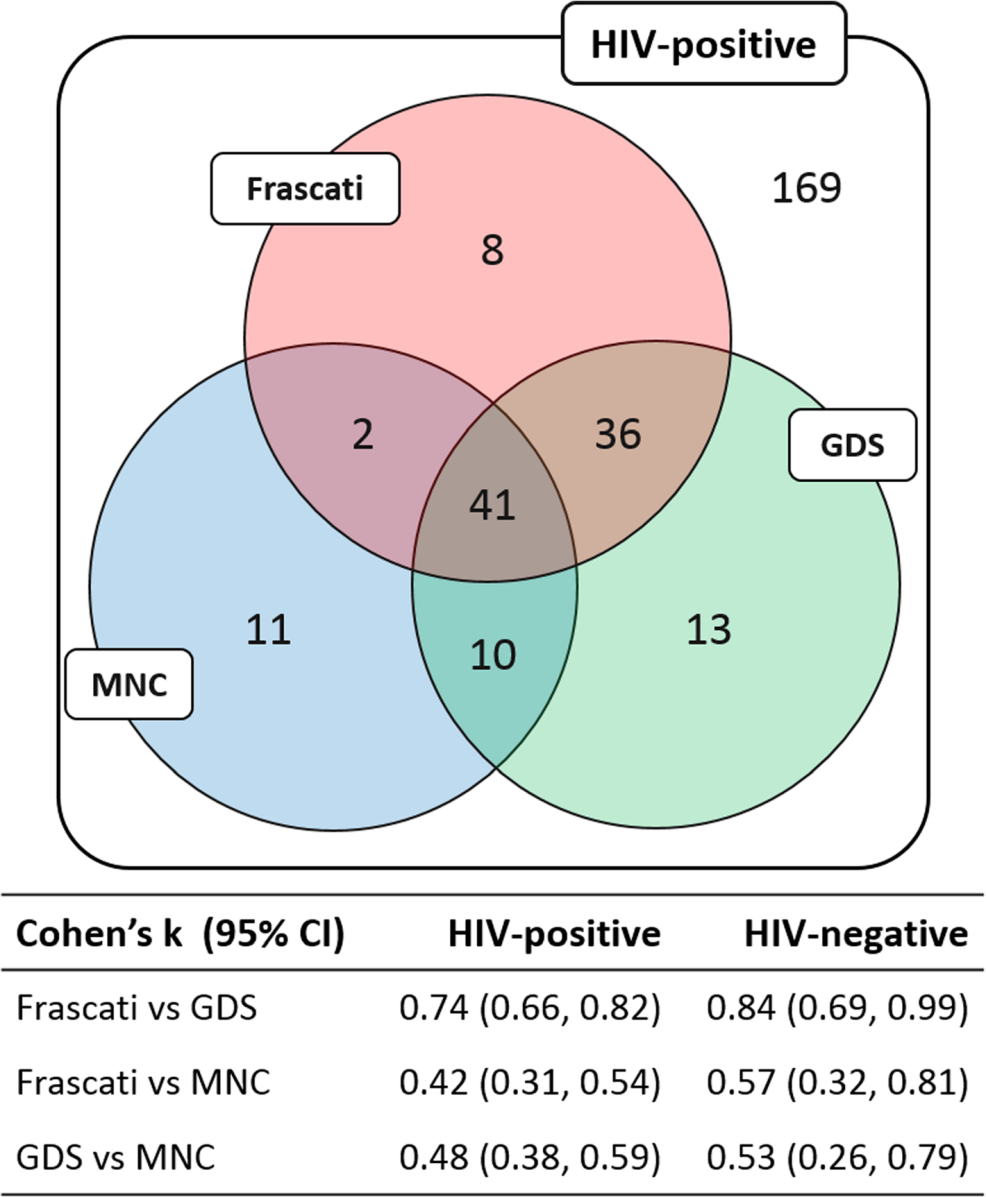
Classification of CI among HIV-positive individuals according to the three criteria and agreement between criteria in HIV-positive and HIV-negative participants

**Fig. 2:**
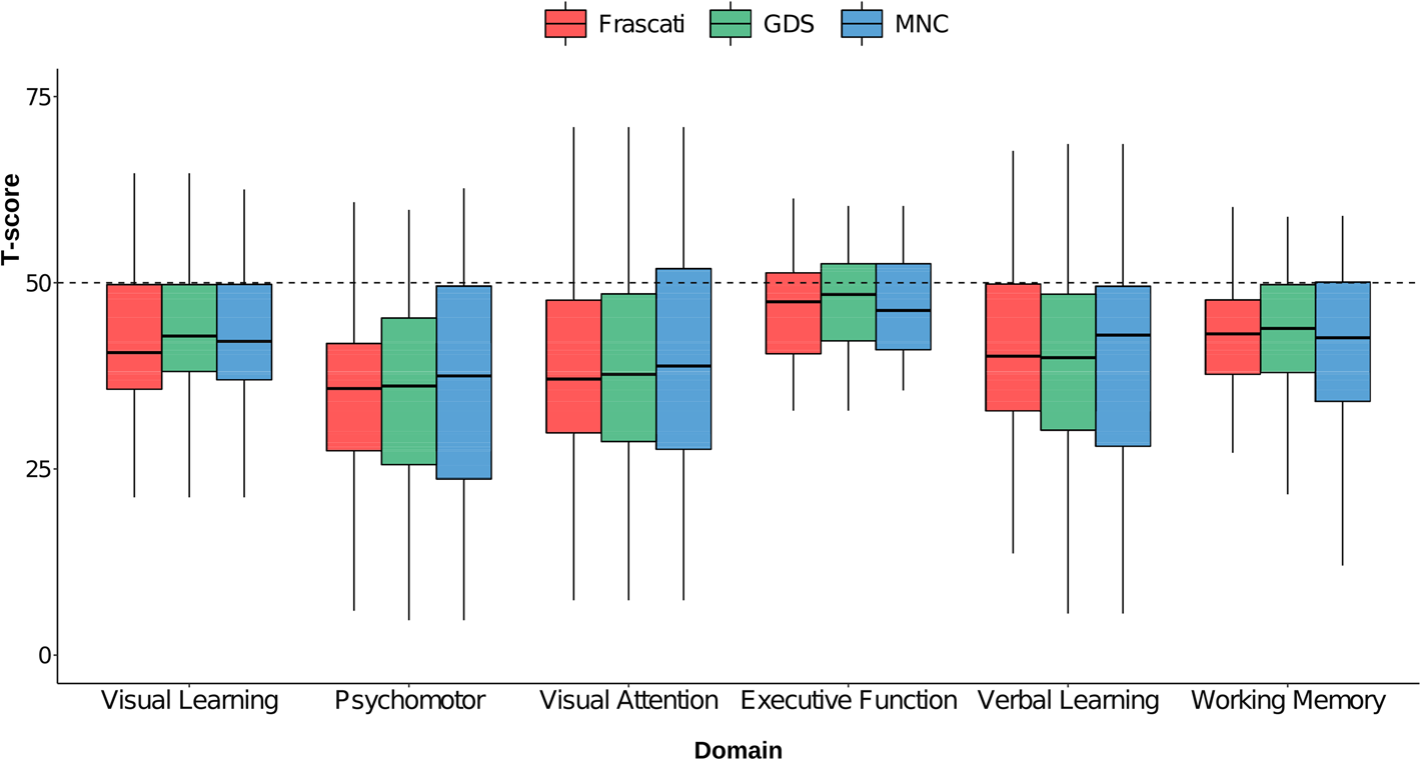
Domain T-scores in HIV-positive individuals classified as cognitively impaired by the three criteria

Overlap in the classification of CI between the three criteria and Cohen’s κ statistics are reported in Fig. 1. Overall 169 (58.3 %) HIV-positive individuals did not meet any of the three definitions of CI while 41 (14.1 %) were classified as cognitively impaired by all the three definitions. Thirty-six (12.4 %) met Frascati and GDS only, 10 (3.4 %) met GDS and MNC only and 2 (0.7 %) met Frascati and MNC only. Frascati and GDS showed a substantial agreement (*κ* = 0.74, *p* < 0.01) while Frascati and MNC, and GDS and MNC showed moderate agreement (*κ* = 0.42 and *κ* = 0.48, respectively; *p* < 0.01 for each). Definitions showed better agreement when tested on the HIV-negative group: *κ* = 0.84 (*p* < 0.01) for Frascati and GDS, *κ* = 0.57 (*p* < 0.01) for Frascati and MNC and *κ* = 0.53 (*p* < 0.01) for GDS and MNC. Cognitive scores in individual domains of HIV-positive individuals meeting each of the three definition were similar (Fig. 2).

### Association between definitions of CI and self-reported cognitive problems

Overall, 14, 15 and 17 HIV-positive individuals did not answer or had missing information on memory loss, reasoning and attention problems, respectively. Among those with complete information, 79 (28.6 %) reported frequent memory loss, 105 (38.2 %) reported reasoning problems and 79 (28.9 %) attention problems; moreover 90 (32.5 %) reported at least two of the three problems. Internal consistency of the three patient-reported measures was excellent with a Cronbach’s α (95 % CI) of 0.9 (0.82, 0.98). A significant association was found between memory loss and all the three definitions of CI (Table 2): using the Frascati criteria, 38 % of subjects with CI reported frequent memory loss, while only 25 % did so among those without CI (*p* = 0.02), with GDS the proportions were 40 and 23 % (*p* < 0.01) for those with and without CI, respectively, and with MNC 41 and 25 % (*p* = 0.02). There was no strong evidence for associations between reasoning problems and self-reported attention problems with definitions of CI, regardless of the definition (Reasoning: *p* = 0.77, *p* = 0.24 and *p* = 0.42 for Frascati, GDS and MNC, respectively; Attention: *p =* 0.59, *p =* 0.07 and *p =* 0.28 for Frascati, GDS and MNC, respectively).

**Table 2:** Frequency distribution (with row percentages) of memory loss, reasoning and attention problems by status of CI according to the three criteria among HIV-positive individuals

	Memory loss, n (%)			Reasoning, n (%)			Attention, n (%)		
	No	Yes	*p*	No	Yes	*p*	No	Yes	*p*
Frascati			0.02			0.77			0.59
CI	50 (62 %)	31 (38 %)		49 (60 %)	32 (40 %)		55 (69 %)	25 (31 %)	
Not CI	147 (75 %)	48 (25 %)		121 (62 %)	73 (38 %)		139 (72 %)	54 (28 %)	
GDS			<0.01			0.24			0.07
CI	56 (60 %)	37 (40 %)		53 (57 %)	40 (43 %)		59 (64 %)	33 (36 %)	
Not CI	141 (77 %)	42 (23 %)		117 (64 %)	65 (36 %)		135 (75 %)	46 (25 %)	
MNC			0.02			0.42			0.28
CI	36 (59 %)	25 (41 %)		35 (57 %)	26 (43 %)		40 (66 %)	21 (34 %)	
Not CI	161 (75 %)	54 (25 %)		135 (63 %)	79 (37 %)		154 (73 %)	58 (27 %)	

14, 15 and 17 HIV-positive individuals had missing information on memory loss, reasoning and attention problems, respectively

### Cognitive function and self-reported symptoms and overlap between definitions of CI

**Fig. 3:**
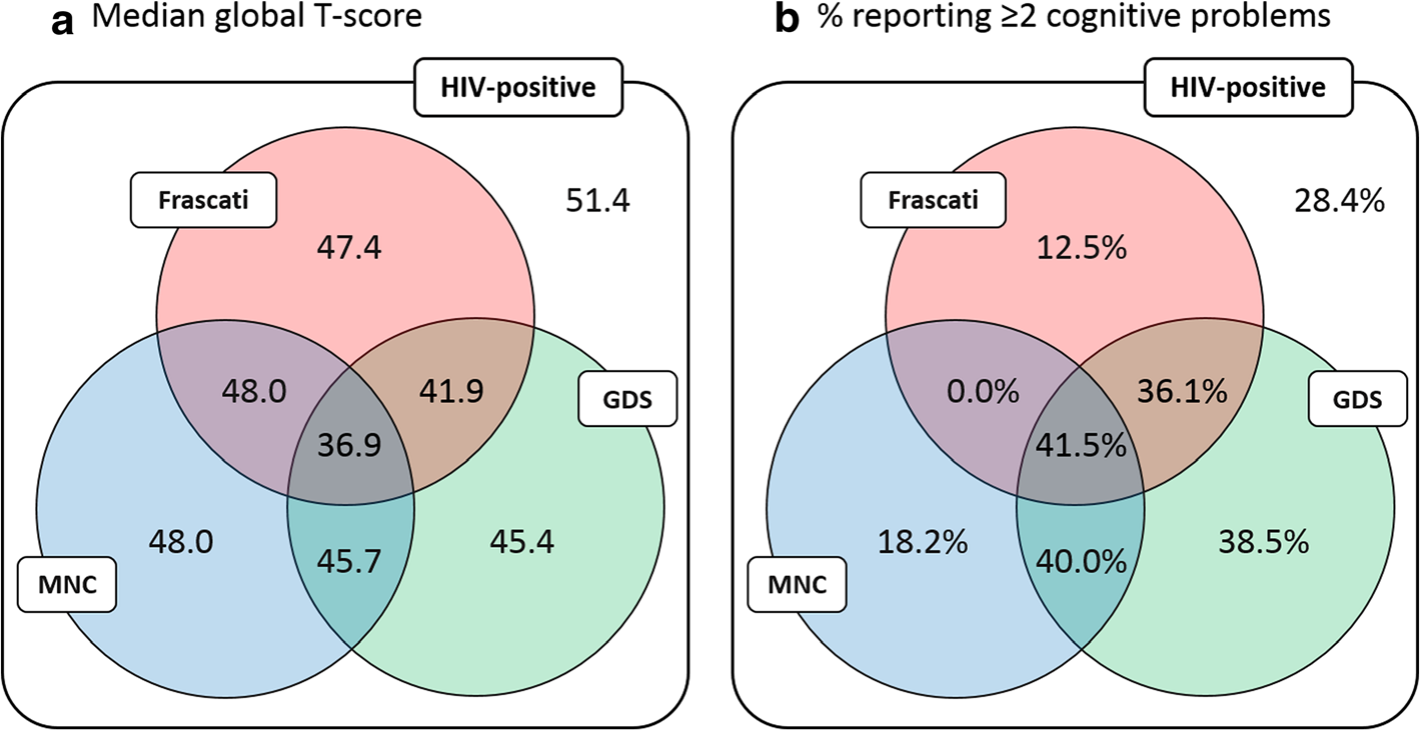
Median global T-score (**a**) and proportion of subjects reporting two or more cognitive problems (**b**) by subset of HIV-positive participants meeting different combinations of the three definitions of CI

Those meeting all three definitions (*n* = 41, 14.1 %) had a low median global T-score (36.9, Fig. 3), indicating poorer cognitive function, and approximately 41.5 % reported at least two of memory loss, reasoning and attention problems. In particular, performances in the psychomotor (median score: 26) and visual attention (33.1) domains were particularly poor. The 36 (12.4 %) subjects with CI according to Frascati and GDS (but not MNC) demonstrated similar cognitive function: 13 of them (36.1 %) reported at least two cognitive problems and the median global T-score was 41.9. On the other hand, the 11 (3.8 %) subjects identified as cognitively impaired only by MNC performed generally better (median global T-score equals to 48.0) and a lower proportion (18.2 %) reported two or more cognitive problems.

## Discussion

HIV-positive individuals exhibit poorer cognitive function when compared to an appropriate HIV-negative control group. Although the difference in the overall cognitive score is statistically significant, this would not be considered clinically meaningful (for T-scores, a 5-point difference is usually considered relevant from a clinical point of view [28]). The prevalence of CI in older HIV-positive individuals may vary from 35 to 22 % depending on the criteria used.

Commonly-used criteria of CI show fair agreement, especially Frascati and GDS. However, this agreement is mainly driven by the ability of criteria to exclude CI in the same set of subjects rather than their ability to identify CI. As expected, subjects meeting all the criteria have generally poorer cognitive function (with particularly poor performances in the psychomotor and visual attention domains) and are more likely to experience cognitive problems. Similar cognitive scores were also observed in subjects classified as impaired by Frascati and GDS (but not MNC).

Consistent with several published studies [14, 15], but contrary to others [12, 13], the associations with self-reported cognitive symptoms are generally poor for all the three definitions of CI considered. Whilst CI, defined with all three criteria, correlate with memory loss, this is not the case for either attention or reasoning problems. These results, based on a cohort of mainly treated and virally-suppressed subjects, shed further light on the association between patient-reported and objective cognitive impairment in the post-ART era. The lack of association found may reflect the pattern of cognitive changes we observed, namely, poorer verbal learning (which may relate to patient reported memory problems) but no significant differences in executive function (reasoning) or working memory (attention). These results suggest a potential lack of a clear relationship between subjective measures of cognitive function and more objective measures based on neuropsychological tests, particularly in those with mild impairment. Several reasons may account for this lack of association, such as the over-reporting of cognitive symptoms and the subjectivity of the EACS questions. Moreover, depressive disorders have been previously reported to affect both subjective and objective cognitive function [29], and can therefore confound the association between the two. We did not co-vary depression in our analyses, but from preliminary analysis, depression did not seem to change the associations between definitions of CI and patient-reported symptoms (data not shown).

A missing answer to questions about cognitive complaints may, in theory, be informative as it may indicate poor understanding or attention. However, almost 95 % of data was complete so it’s unlikely that the exclusion of this small group of individuals from analyses has introduced substantial bias. Although ART prescribing was in line with national guidelines, 3.1 % of enrolled HIV-positive individuals were not receiving suppressive antiretroviral therapy at study entry. This is justified by the aim of the study of recruiting a ‘real-world’ sample of PLWH in UK and Ireland. Nonetheless those not receiving ART had a median CD4+ cell count of 664 cells/μL, which makes it unlikely that lack of suppression of HIV replication in a minority of the sample has led to substantial bias to our findings.

Given the lack of a gold standard in defining CI, it is difficult to ascertain the validity of different definitions. An optimal definition of CI would capture subjects with the lowest cognitive performance scores and the greatest number of cognitive symptom complaints. In our study we observed the lowest overall cognitive score in subjects meeting all three definitions (median global T-score of 36.9 for those meeting Frascati, MNC and GDS). Moreover, in this group of subject, the number reporting cognitive complaints was highest (41.5 %). Given these findings, we consider further work to assess longitudinal outcomes in HIV-positive individuals meeting this definition of CI within the POPPY study, compared to other definitions of cognitive impairment, is justified.

Other definitions of cognitive impairment in PLWH, such as the Frascati criteria, have been criticised for overcalling the number of PLWH with cognitive deficits [11]. On the converse argument, the Frascati criteria has attempted to define the presence of cognitive deficits prior to the onset of clinical symptomatology and classifies large numbers of subjects with CI. The rationale behind such criteria being interventions at an early stage of disease, if effective, may prevent the onset of clinically apparent conditions. However, to date, longitudinal data both in the HIV-field and in other neurodegenerative diseases have not provided convincing evidence for the diagnosis of a pre-morbid cognitive state [30, 31].

There are several other problems when utilising non-stringent definitions of CI. Firstly, as large numbers of patients will meet a diagnostic criteria, unnecessary anxiety for patients could be created. Secondly, non-stringent definitions will include subjects with cognitive impairment but will also include subjects who do not have cognitive impairment. Within interventional trials, this may lead to the null hypothesis being proven when in fact an intervention does work. By including patients without a disease state in an interventional study, the results of the study may suggest an effective interventions has no effect whereas, if the effective intervention was trialled in the diseased population, an effect may be observed.

## Conclusions

Commonly-used criteria of CI show fair agreement, especially in identifying subjects without CI. However their association with patient-reported symptoms is generally weak and we hypothesise that our POPPY Study definition of cognitive impairment may be a pragmatic approach to adopt at the current time. Our definition, by defining subjects with the highest rates of symptomatology and the lowest global cognitive score is likely to capture patients with true pathological cognitive impairment and requires validation within other HIV-cohorts and in longitudinal settings.

## Additional file

Additional file 1: Table S1. Cognitive tests administered by cognitive domain. (DOCX 13 kb)

## Abbreviations

ART: Antiretroviral therapy
CI: Cognitive impairment
EACS: European AIDS Clinical Society
GDS: Global deficit score
HAND: HIV-associated neurocognitive disorder
MNC: Multivariate normative comparison
PLWH: People living with HIV
POPPY: Pharmacokinetic and Clinical Observations in People Over Fifty
IQR: Interquartile range
