# Supplementary material for: Defining cognitive impairment in people-living-with-HIV: the POPPY study
Source: BMC Infect Dis. 2016 Oct 28;16:617. doi: 10.1186/s12879-016-1970-8 (PMC5084371; doi:10.1186/s12879-016-1970-8)
Supplement: Additional file 1: Table S1. — Cognitive tests administered by cognitive domain. (DOCX 13 kb) [file 12879_2016_1970_MOESM1_ESM.docx]

Table S1: Cognitive tests administered by cognitive domain

| **Cognitive domain** | **Tests** |
| --- | --- |
| Visual Learning | - Continuous paired associate learning test - Groton Maze Learning test – delayed recall - One card learning task |
| Psychomotor function | - Detection task |
| Visual Attention | - Identification task |
| Executive Function | - Groton Maze Learning test - Set shifting task |
| Verbal Learning | - International Shopping list task - International Shopping list task – delayed recall |
| Working Memory/Attention | - One back task - Two back task |
